# Deciphering the roles of non-coding RNAs in liposarcoma development: Challenges and opportunities for translational therapeutic advances

**DOI:** 10.1016/j.ncrna.2024.11.005

**Published:** 2024-11-15

**Authors:** Zhi Xiong Chong, Wan Yong Ho, Swee Keong Yeap

**Affiliations:** a NUS Centre for Cancer Research, Yong Loo Lin School of Medicine, National University of Singapore, 14 Medical Drive, 117599, Singapore; b Cancer Science Institute of Singapore, National University of Singapore, 14 Medical Drive, 117599, Singapore; c Faculty of Science and Engineering, University of Nottingham Malaysia, Jalan Broga, 43500, Semenyih, Selangor, Malaysia; d China-ASEAN College of Marine Sciences, Xiamen University Malaysia, 43900, Sepang, Selangor, Malaysia

**Keywords:** Liposarcoma, ncRNAs, miRNAs, lncRNAs, Diagnosis, Prognosis

## Abstract

Liposarcoma is one of the most prevalent forms of soft tissue sarcoma, and its prognosis is highly dependent on its molecular subtypes. Non-coding RNAs (ncRNAs) like microRNAs (miRNAs) and long non-coding RNAs (lncRNAs) can bind various cellular targets to regulate carcinogenesis. By affecting the expressions and activities of their downstream targets post-transcriptionally, dysregulations of miRNAs can alter different oncogenic signalling pathways, mediating liposarcoma progression. On the contrary, lncRNAs can sponge miRNAs to spare their downstream targets from translational repression, indirectly affecting miRNA-regulated oncogenic activities. In the past 15 years, multiple fundamental and clinical research has shown that different ncRNAs play essential roles in modulating liposarcoma development. Yet, there is a lack of an effective review report that could summarize the findings from various studies. To narrow this literature gap, this review article aimed to compare the findings from different studies on the tumour-regulatory roles of ncRNAs in liposarcoma and to understand how ncRNAs control liposarcoma progression mechanistically. Additionally, the reported findings were critically reviewed to evaluate the translational potentials of various ncRNAs in clinical applications, including employing these ncRNAs as diagnostic and prognostic biomarkers or as therapeutic targets in the management of liposarcoma. Overall, over 15 ncRNAs were reported to play essential roles in modulating different cellular pathways, including apoptosis, WNT/β-catenin, TGF-β/SMAD4, EMT, interleukin, and YAP-associated pathways to influence liposarcoma development. 28 ncRNAs were reported to be upregulated in liposarcoma tissues or circulation, whereas 11 were downregulated, making them potential candidates as liposarcoma diagnostic biomarkers. Among these ncRNAs, measuring the tissues or circulating levels of miR-155 and miR-195 was reported to help detect liposarcoma, differentiate liposarcoma subtypes, and predict the survival and treatment response of liposarcoma patients. Overall, except for a few ncRNAs like miR-155 and miR-195, current evidence to support the use of discussed ncRNAs as biomarkers and therapeutic targets in managing liposarcoma is mainly based on a single-center study with relatively small sample sizes or cell-based studies. Hence, more large-scale multi-center studies should be conducted to further confirm the sensitivity, specificity, and safety of ncRNAs as biomarkers and therapeutic targets. Instead of furthering investigation to confirm the translational values of all the discussed ncRNAs, which can be time- and cost-consuming, it would be more practical to focus on a few ncRNAs, including miR-155 and miR-195, to evaluate if they are sensitive and safe to be used as liposarcoma biomarkers and therapeutic agents or targets.

## Graphical abstract

**Figure:**
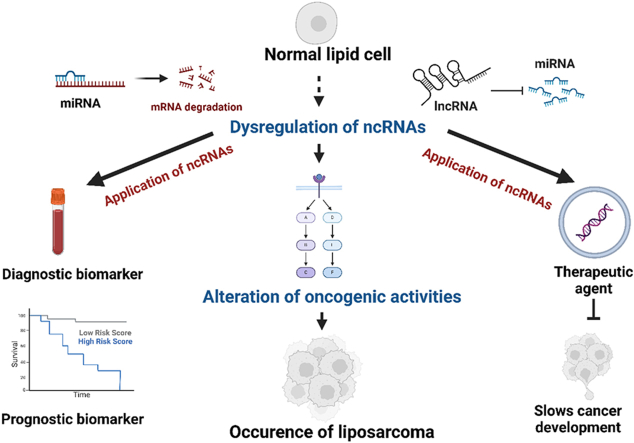

## Abbreviations

AKT: Protein kinase B
AUC: Area under curve
BCL: B-cell leukemia
circRNA: Circular RNA
CK1α: Casein kinase 1α
CRKL: CRK-like proto-oncogene
DDLPS: Dedifferentiated liposarcomas
DFS: Disease-free survival
DHX9: DExH-box helicase 9
EGFR: Epidermal growth factor receptor
eIF4A3: Eukaryotic translation initiation factor
EMT: Epithelial-to-mesenchymal transition
FAK: Focal adhesion kinase
FAM98A: Family with sequence similarity 98 member A
FC: Fold-change
FOXM1: Forkhead box protein M1
HIF: Hypoxia inducible factor
HOXA: Homeobox protein
IGF1R: Insulin-like growth factor 1 receptor
IL: Interleukin
lncRNA: Long non-coding RNA
MAPK: Mitogen-activated protein kinase
MDM2: Mouse double minute 2 homolog
miRNA: MicroRNA
MMP: Matrix metalloproteinase
NA: No information available
ncRNA: Non-coding RNA
NFATC3: Nuclear factor of activated T cells 3
NF-Kβ: Nuclear factor-kappa beta
OS: Overall survival
OSBP: Oxysterol-binding protein
PAI-1: Plasminogen activator inhibitor-1
PDGFR: Platelet-derived growth factor receptor
PI3K: Phosphoinositide 3-kinase
PLK1: Polo-like kinase 1
PRC1: Protein regulator of cytokinesis 1
RCBTB1: Regulator of chromosome condensation and BTB domain-containing protein 1
SAMD4B: Sterile alpha motif domain containing 4B
SMAD: Suppressor of mothers against Decapentaplegic
SOX9: SRY-box transcription factor 9
SRPK1: Serine-arginine protein kinase 1
STAT3: Signal transducer and activator of transcription 3
TAM: Tumour-associated macrophage
TCL1A: T-cell leukemia/lymphoma protein 1A
TGF: Transforming growth factor
THBS2: Thrombospondin 2
TNM: Tumour size, nodal involvement, and metastases
TOP2A: Topoisomerase 2-alpha
TP: Tumour-promoter
TS: Tumour-suppressor
UTR: Untranslated region
WDLPS: Well-differentiated liposarcoma
WHO: World Health Organization
YAP: Yes-associated protein
YY1: Yin Yang 1

## 1 Introduction

As one of the most frequently diagnosed soft tissue sarcomas, liposarcoma makes up around 15 %–20 % of all detected soft tissue malignancies [[1], [2], [3]]. In other words, approximately one out of five diagnosed soft tissue cases will be a liposarcoma case. Depending on the histological subtypes (Table 1), some liposarcoma can be chemoresistant and can have a poor prognosis with moderate to high recurrence risk [[4], [5], [6]]. An example is dedifferentiated liposarcoma (DDLPS), which constitutes around one-fifth of all diagnosed liposarcoma cases [4]. This type of liposarcoma can metastasize to other organs, such as the lung [7], and the 5-year mortality rate of DDLPS patients can be as low as 30 % [8]. This makes DDLPS the liposarcoma with the lowest survival rate compared to other liposarcoma subtypes [2]. Surgical resection is the key treatment option for most liposarcoma cases [6,9]. However, liposarcoma tends to recur when the surgical resection is incomplete [9]. Additionally, some liposarcoma subtypes like DDLPS respond poorly to chemotherapeutic agents [5]. To increase the survival rate of liposarcoma patients, clinician scientists are always looking into alternative therapeutic options that could help completely eradicate liposarcoma in cancer patients [[10], [11], [12]] and example of the newer therapeutic approach is the use of non-coding RNAs (ncRNAs) like microRNAs (miRNAs), long non-coding RNAs (LncRNAs), or circular RNAs (circRNAs).

**Table 1:** Different subtypes of liposarcoma and their prognosis.

	Myxoid liposarcoma	Well-dedifferentiated liposarcoma (WDLPS)	Dedifferentiated liposarcoma (DDLPS)	Pleomorphic liposarcoma	Myxoid pleomorphic liposarcoma
**Characteristics**	Continuum between pure myxoid/round cell morphology	Locally aggressive, well-differentiated adipocytic sarcoma	Malignant, progressing from well-differentiated to non-lipogenic tumour	Aggressive, undifferentiated sarcoma	Combination of myxoid & pleomorphic features
**Constitutes how many % of liposarcoma**	20–30 %	40–50 %	15–20 %	5–10 %	15 %
**Treatment options**	Surgery, neoadjuvant/adjuvant radiotherapy, chemotherapy, targeted therapy	Surgery with negative margins	Neoadjuvant radiotherapy, surgery, targeted therapy	Surgery, post-operative radiotherapy, chemotherapy	Surgery with negative margins, benefits of chemotherapy & radiotherapy not well-confirmed
**Median survival/mortality rate**	5-year survival rate: 80–90 % for pure myxoid subtype & 20–25 % for tumour containing high-grade round cells	Median time to death: 6–11 years	Mortality rate: ∼30 %	5-year survival rate: 60 %	Median time to death: 2 years
**References**	[1,2,4,6,26,59]	[1,2,4,5,26]	[2,4,5,8,26]	[2,4,5,26]	[4,60,61]

MiRNAs are short, non-coding RNAs made up of 16–23 nucleotides [13], and they can regulate the expressions and activities of various oncogenic targets, affecting the carcinogenesis process [14,15]. By interacting with the 3′- or 5′-untranslated regions (UTRs) of their downstream targets, miRNAs can repress their translational activities, reducing the expressions and activities of these downstream targets [16,17]. When miRNAs suppress the expression of tumour-promoting proteins, this will decrease the carcinogenesis process in cancer cells [18], and the reverse phenomenon will occur when miRNAs repress the cellular level of tumour-suppressing proteins [19]. Long non-coding RNAs (LncRNAs), on the other hand, usually contain at least 200 nucleotides [20], and they can bind and sponge miRNAs, sparing the downstream targets of miRNAs from translational repression or decay [21,22]. Like lncRNAs, circRNAs could also act as miRNA sponges, relieving the mRNA targets of miRNAs from translational inhibition [23]. Therefore, dysregulations of ncRNAs in cancer cells can alter the cellular expression and activities of various cellular oncogenic proteins, resulting in enhanced tumour progression [24,25]. Besides conventional surgical, chemotherapy, radiotherapy, and targeted therapy [4,26], understanding how ncRNAs regulate cancer pathways can help scientists find new cures to treat liposarcoma [[27], [28], [29]]. For example, miR-193b has been reported to suppress the activity of the Hippo pathway to slow liposarcoma cell growth [30], and increasing its expression could potentially help decrease liposarcoma progression. On the contrary, LINC00423 was reported to suppress the oncogenic mitogen-activated protein kinase (MAPK) pathway [28], and upregulating it could serve as a strategy to slow liposarcoma growth. Other than having potential translational therapeutic potential, ncRNAs could also be potentially used to diagnose and predict the prognosis of liposarcoma patients [[31], [32], [33]]. For instance, miR-26a-2 is upregulated by around 10-fold in both liposarcoma tissues and cell lines [34,35], making it a potential translatable biomarker in diagnosing liposarcoma. Increased expression of miR-26a-2 was also demonstrated to correlate to low survival among liposarcoma patients [34], making this miRNA a suitable candidate to predict the clinical outcomes of liposarcoma patients.

Over the past 15 years, emerging evidence and multiple studies (Table 2, Table 3) have shown that various ncRNAs can regulate liposarcoma development. These studies span from fundamental pre-clinical to clinical studies, and they demonstrated that multiple ncRNAs could modulate the activities of different oncogenic signalling activities, moderating liposarcoma progression. Additionally, various ncRNAs were dysregulated in liposarcoma tissues or circulation. However, there is a gap in the literature as there is currently a lack of an effective review report that could summarize the regulatory roles and translational values of various ncRNAs in liposarcoma. To fill this literature gap, this review aims to compare the key findings reported in different studies on the potential tumour-regulatory roles of various ncRNAs and to understand how different ncRNAs can control liposarcoma progression mechanistically. Subsequently, the findings from multiple studies will be critically reviewed to evaluate if the discussed ncRNAs have translational potential, including the possibility of being employed as biomarkers or therapeutic targets in managing liposarcoma. With this, it is hoped that the review can provide an up-to-date summary of the progress of the roles of ncRNAs in regulating the development of liposarcoma and its translational potential in the near future.

**Table 2:** Roles of miRNAs in regulating the development of liposarcoma (n = 14).

miRNA	Study Design	Cell/Circulating Level (FC)	TP/TS	Downstream Targets/Pathways	Effects	References
miR-26a-2	*In vitro*	↑ Cancer cell lines (∼10-fold)	TP	↓ HOXA5	↓ Apoptosis	[35]
miR-26a-2	Clinical & *in vitro*	↑ Cancerous tissues (∼10-fold)	TP	↓ RCBTB1	↑ Proliferation & migration ↓ Differentiation & apoptosis	[34]
miR-155	Clinical, *in vitro* & *in vivo*	↑ Cancerous tissues (∼7.5-fold) & cancer cell lines (∼20-60-fold)	TP	↓ CK1α	↑ Cyclin D1 expression, Β-catenin signalling & tumour growth	[70]
miR-135b	Clinical, *in vitro* & *in vivo*	↑ Cancerous tissues (∼20-fold) & cancer cell lines (∼10-15-fold)	TP	↓ THBS2	↑ Invasion & migration in myxoid liposarcoma	[75]
miR-215-5p	*In vitro*	↑ Cancer cell lines (∼25-fold)	TP	↑ MDM2	↑ Proliferation & invasion ↓ Apoptosis	[82]
miR-25-3p & miR-92a-3p	Clinical & *in vitro*	↑ Exosome (2.5-3-fold)	TP	↑ IL-6	↑ IL-6-mediated inflammation ↑ Growth, invasion & migration	[85]
miR-143	Clinical & *in vitro*	↓ Cancerous tissues (3.3–7.9-fold) & cancer cell lines (∼10-fold)	TS	↓ BCL-2, TOP2A, PRC1 & PLK1	↓ Proliferation ↑ Apoptosis in DDLPS & WDLPS	[90]
miR-133a	Clinical, *in vitro* & *in vivo*	↓ Cancerous tissues (40-fold) & cancer cell lines (2-5-fold)	TS	NA	↓ Proliferation, cell cycle progression & glycolysis ↑ Oxidative phosphorylation	[128]
miR-193b	Clinical & *in vitro*	↓ Cancerous tissues (6-40-fold) & cancer cell lines (2-5-fold)	TS	↓ PDGFRβ, SMAD4 & YAP1	↓ Proliferation ↑ Adipogenesis & differentiation	[30]
miR-193b	Clinical, *in vitro* & *in vivo*	↓ Cancerous tissues (3.9-fold) & cancer cell lines (∼2-10-fold)	TS	↓ CRKL & FAK	↓ Proliferation ↑ Adipogenesis & differentiation	[98]
miR-195	Clinical, *in vitro* & *in vivo*	↓ Cancerous tissues (∼2-fold)	TS	↓ OSBP	↓ Growth & migration ↑ Apoptosis	[106]
miR-486	*In vitro*	NA	TS	↓ PAI-1	↓ Growth in human myxoid liposarcoma	[114]
miR-210-3p & miR-485-5p	*In vitro*	NA	TS	↓ HIF-3α	↓ Hypoxic-signalling pathway	[119]
miR-145 & miR-451	Clinical & *in vitro*	↓ Cancerous tissues (∼2-5-fold)	TS	NA	↓ Growth & cell cycle progression ↑ Apoptosis	[33]

NA: No information available; ↑ Increase; ↓ Decrease; TP: Tumour-promoter; TS: Tumour-suppressor.

**Table 3:** Roles of lncRNAs in modulating the progression of liposarcoma (n = 3).

LncRNA	Study Design	Cell/Circulating Level (FC)	TP/TS	Downstream Targets/Pathways	Effects	References
TODL	Clinical, *in vitro* & *in vivo*	↑ Cancerous tissues (∼5-20-fold) & cancer cell lines (∼80-180-fold)	TP	FOXM1 (Binding)	↑ Proliferation, cell cycle progression, EMT & migration ↓ Adipogenesis & differentiation in DDLPS	[31]
PILRLS	Clinical & *in vitro*	↑ Cancerous tissues (∼5-fold)	TP	TCL1A (Binding)	↑ MDM2 ↓ p53 to promote proliferation in retroperitoneal liposarcoma	[27]
LINC00423	Clinical, *in vitro* & *in vivo*	↓ Cancerous tissues (∼1-2-fold)	TS	NFATC3 (Destabilization)	↓ MAPK to slow retroperitoneal liposarcoma growth	[28]

↑ Increase; ↓ Decrease; TP: Tumour-promoter; TS: Tumour-suppressor.

## 2 Types of ncRNAs and their roles in carcinogenesis

Generally, ncRNAs can be divided into three main groups, namely miRNAs [36,37], lncRNAs [38,39], and circRNAs [40,41]. Each type of ncRNA could play a vital role in regulating cancer development by controlling the cellular levels or activities of various oncogenic and tumour-inhibiting proteins [[42], [43], [44]].

### 2.1 miRNAs

As a single-stranded, small, non-coding RNA consisting of 16–23 nucleotides [13], miRNA is known to modulate cancer progression [45]. Generally, tumour-regulating miRNA can be divided into tumour-promoting and tumour-suppressing miRNA [46]. When miRNA binds to the untranslated region of its mRNA target, it can induce translational inhibition or decay, leading to repression of the target expression [16,17]. Tumour-promoting miRNA accelerates cancer development by decreasing the expression or activity of tumour-suppressing proteins [44,47]. For instance, miR-1205 could suppress the expression of cyclin-dependent kinase 3 (CDK3), a cellular protein that binds SNAIL to induce its degradation in breast cancer cells [44]. SNAIL is critical in promoting epithelial-to-mesenchymal transition (EMT), and EMT is important in accelerating breast cancer cell metastases [44]. When CDK3 expression is repressed by miR-1205, SNAIL accumulates intracellularly to promote EMT and breast cancer metastases [44]. Another example of tumour-promoting miRNA is miR-10a-5p, which can decrease the expression of ubiquitin-conjugating enzyme E2I (UBE2I), an essential protein involved in controlling the ubiquitination process of various cellular proteins [47]. Suppression of UBE2I then promotes cervical cancer progression, and its dysregulation can also precipitate the worsening of hepatocellular carcinoma [47]. On the other hand, tumour-suppressing miRNA can inhibit the expression of oncogenic proteins or increase the cellular level of tumour-inhibiting proteins, leading to repression of tumour development [48,49]. For example, miR-323a-3p can bind and repress the expression of signal transducer and activator of transcription 3 (STAT3) to inhibit tumour progression in neuroblastoma [49]. On the contrary, miR-3938 can downregulate the expression of mouse double minute 2 homolog (MDM2) to increase the expression of tumour-suppressor protein p53, leading to inhibition of glioblastoma progression [48]. Besides binding to untranslated regions, miRNAs can also bind to gene promoters to increase cellular protein expression [50]. An example is miR-181d, which could bind to the TATA-box motifs of the c-MYC gene promoter to enhance its activities, increasing c-MYC gene expression [51].

### 2.2 lncRNAs

Compared to miRNAs, lncRNAs are single-stranded but longer in length and contain more than 200 nucleotides [52]. LncRNA can regulate tumour progression by binding and sponging miRNA, sparing the miRNA targets from translation repression [38,39]. For example, lncRNA MLETA1 was reported to bind miR-186-5p and miR-497-5p in non-small cell lung cancer [38]. The binding prevents the translational inhibition of epidermal growth factor receptor (EGFR) and insulin-like growth factor 1 receptor (IGF1R), which both are essential cellular oncogenic proteins that drive carcinogenesis in human cancers such as lung cancer [38]. Besides acting as a tumour-promoter, lncRNAs such as lncRNA GAS5 can act as a tumour suppressor by sponging tumour-enhancing miRNA, e.g., miR-23 in ovarian cancer [39]. When unbound, miR-23a can bind and induce the translational repression of WT1, a critical tumour suppressor in ovarian cells [39]. The sponging of miR-23a by lncRNA GAS5 relieves WT1 from translational suppression, allowing it to exert its tumour inhibiting function to halt tumour progression [39]. Conversely, lncRNA can also competitively bind cellular proteins to regulate cellular activities like EMT [43]. For instance, lncRNA CARMN can competitively bind DExH-box helicase 9 (DHX9) to inhibit the transcriptional activation of matrix metalloproteinase 2 (MMP2), a critical regulator of EMT and metastases [43]. Reducing cellular MMP2 then decreases breast cancer cell migration and metastases [43].

### 2.3 circRNAs

CircRNA is a single-stranded and non-coding RNA characterized by a covalently closed structure [40,41]. It does not contain the 5′ cap and 3’ poly(A) tail usually found in the mRNA of mammalian cells, and it is formed through a back-splicing process that occurs inside the cell nucleus [40,41]. CircRNAs can sponge miRNAs like what lncRNAs do to affect cellular and molecular activities [53,54]. CircPTPRF was demonstrated to competitively bind miR-1208 to release Yin Yang 1 (YY1) from translational blocking, elevating the cellular level of this transcriptional repressor in glioblastoma [54]. This resulted in the progression and worsening of glioblastoma and suggested that circPTPRF could act as a tumour-repressing circRNA in glioblastoma [54]. On the other hand, circLIFR can sponge miR-429 to increase the expression of tissue inhibitor of metalloproteinase 2 (TIMP2) in papillary thyroid cancer [53], a crucial regulator of EMT and cancer metastases [55]. As a result, this caused the suppression of EMT, and the progression of papillary thyroid cancer implied that circLIFR could serve as a tumour repressing circRNA in thyroid cancer [53]. As an oncogenic protein accelerating the progression of multiple human cancers [56], c-MYC dysregulation is positively correlated with poor clinical outcomes among cancer patients [57]. Circ_0000467 was reported to be capable of binding eukaryotic translation initiation factor 4A3 (eIF4A3) in colorectal cancer cells to prevent nuclear translocation of eIF4A3 [42]. Additionally, the circRNA can serve as a scaffold molecule to link eIF4A3 and c-MYC mRNA in the cytoplasm, promoting c-MYC translation [42]. This resulted in the accelerated tumour growth and metastases of colorectal cancer cells, suggesting that circ_0000467 plays a tumour-promoting role by binding and interacting with cellular protein [42].

## 3 Classification of liposarcoma and prognosis of different liposarcoma subtypes

In 2020, the World Health Organization (WHO) classified liposarcoma into five subtypes (Table 1), including myxoid liposarcoma, well-differentiated liposarcoma (WDLPS), dedifferentiated liposarcoma (DDLPS), pleomorphic liposarcoma, and myxoid pleomorphic liposarcoma [3,58]. The prognosis of liposarcoma greatly depends on the molecular subtypes, and tailoring treatment strategies based on different subtypes is crucial in achieving disease remission and preventing recurrence [3,26]. Myxoid liposarcoma makes up around 20 % of all liposarcoma cases [1,26]. This type of liposarcoma has a morphological continuum spanning from pure myxoid to pure round cell appearance [26]. As the disease progresses, more round cells can be found in the tumour tissues, associated with a more aggressive phenotype and poor prognosis [1,2,26]. Surgery, neoadjuvant radiotherapy, and chemotherapy are recommended treatment options for myxoid liposarcoma [6,26,59]. Besides, targeted therapy like immune checkpoint inhibitors has also been used in eradicating myxoid liposarcoma [4,26]. The 5-year survival rate of patients having pure myxoid liposarcoma can be as high as 90 %, while the 5-year survival rate of patients can decrease to below 50 % if they contain high-grade round tumour cells [26].

WDLPS is the most seen liposarcoma, making up around 45 % of all diagnosed liposarcoma cases [1,26]. As a locally aggressive and well-differentiated tumour, WDLPS is usually presented with focal nuclear atypia in stromal and fat cells [4,26]. This type of liposarcoma tends to recur locally and is usually chemoresistant [5,26]. Hence, thorough surgery resection with a negative margin is one of the gold-standard therapeutic approaches to eliminate this cancer [4,26]. Once diagnosed with WDLPS, the median survival of the patients is 6–11 years [26]. When WDLPS progresses to become a non-lipogenic tumour, it becomes dedifferentiated and is now called DDLPS [26]. Compared to WDLPS, DDLPS can have a higher rate of recurrence and metastases [8,26]. DDLPS makes up around 15–20 % of all liposarcoma cases [4], and a study has reported that this kind of liposarcoma has the lowest survival rate compared to other subtypes of liposarcoma [2]. The 5-year mortality rate of DDLPS patients is reported to be around 30 % [8]. Like WDLPS, DDLPS is relatively resistant to chemotherapy [5], and surgical resection is a crucial treatment option for this liposarcoma subtype [4]. However, if complete surgical resection cannot be performed, the risk of recurrence of DDLPS will be high [4]. Therefore, other treatment options for DDLPS include radiotherapy and targeted therapy [26].

Pleomorphic liposarcoma is a rare form of liposarcoma, constituting 5–10 % of all liposarcoma cases [2,26]. This liposarcoma subtype is characterized by undifferentiated cells and pleomorphic lipoblasts [4]. Unlike WDLPS and DDLPS, pleomorphic liposarcoma is generally chemosensitive and responds well to cytotoxic drugs [5], and the treatment options for pleomorphic liposarcoma include surgery, chemotherapy, and adjuvant radiotherapy [4,26]. The 5-year survival rate for patients with pleomorphic liposarcoma is 60 % [26]. On the other hand, myxoid pleomorphic liposarcoma makes up around 15 % of all liposarcoma cases, and it contains the features of myxoid and pleomorphic liposarcoma [60]. Compared to pleomorphic liposarcoma, the prognosis of myxoid pleomorphic liposarcoma is lower [60,61]. A study has reported that the median time to death for patients with pleomorphic liposarcoma is around six years [61]. In comparison, the median time to death for patients with myxoid pleomorphic liposarcoma is just around two years [61]. The currently recommended treatment options for myxoid pleomorphic liposarcoma include complete surgical resection, and the benefits of radiotherapy and chemotherapy in eradicating this sarcoma are still not clearly established [4].

## 4 Roles of miRNAs in the development of liposarcoma

### 4.1 Tumour-promoting miRNAs in liposarcoma

**Fig. 1:**
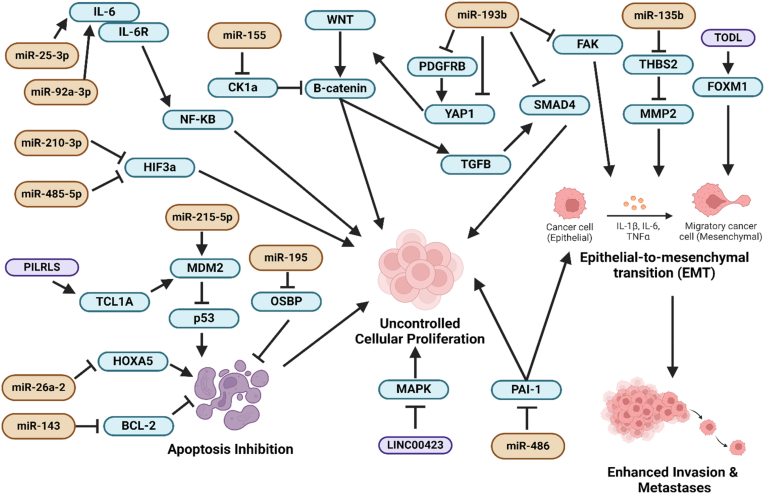
Interaction of various ncRNAs in modulating the progression of liposarcoma. MDM2 plays a crucial role in suppressing p53-mediated apoptosis activities, and miR-215-5p and lncRNA PILRLS can increase MDM2 activity to inhibit apoptosis, promoting liposarcoma growth [27,82]. MiR-26a-2 can repress the expression of pro-apoptotic protein, HOXA5, to increase uncontrolled cellular proliferation in liposarcoma [35]. BCL-2 and OSBP are two anti-apoptotic proteins that miR-143 and miR-195, respectively, can target, and their suppressions will lead to apoptosis induction in liposarcoma cells [90,106]. MiR-193b can target various downstream targets, including PDGFRβ, YAP1, SMAD4, and FAK, to downregulate oncogenic WNT/β-catenin and TGFβ/SMAD pathways to block liposarcoma progression [30,98]. Besides miR-193b, miR-155 can also control the WNT/β-catenin signalling activities by downregulating CK1α, enhancing the nuclear translocation of β-catenin to increase liposarcoma growth [70]. MiR-25-3p and miR-92a-3p can increase the expression of IL-6 in TAM to enhance IL-6-mediated inflammation, proliferation, and metastases in liposarcoma cells [85]. Hypoxia is a critical cellular activity that promotes cellular growth, and miR-210-3p and miR-485-5p can downregulate hypoxia activity in liposarcoma cells by inhibiting HIF-3α [119]. The activated MAPK pathway is known to drive tumourigenesis, and LINC00423 can suppress MAPK activities to reduce cellular growth in liposarcoma [28]. EMT is an essential cellular process that predisposes to cancer metastases, and miR-135b can inhibit THBS2 to increase the expression of MMP2, promoting the breakdown of the tissue basement membrane to accelerate liposarcoma invasion and metastases [75]. PAI-1 also plays a vital role in promoting cancer cell growth and migration, and miR-486 can target PAI-1 to inhibit the growth of myxoid liposarcoma [114]. LncRNA TODL can stabilize FOXM1, contributing to increased EMT and migratory activities in liposarcoma tissues [31]. The diagram was constructed using Biorender (https://app.biorender.com/gallery).

**Fig. 2:**
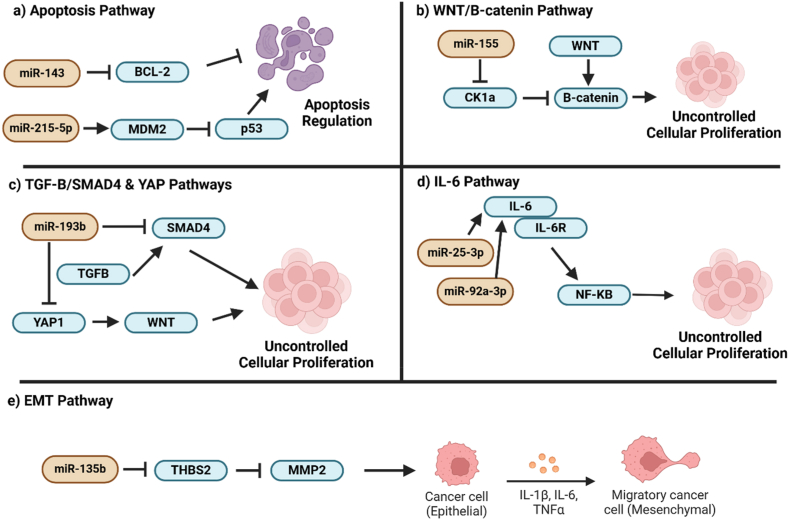
**(a)** MiR-143 can target BCL-2 to promote apoptosis [90], whereas miR-215-5p can increase MDM2 activity to decrease p53-mediated apoptotic activity, resulting in increased liposarcoma proliferation [82]. **(b)** By suppressing CK1α expression, miR-155 can upregulate the WNT/β-catenin pathway to promote liposarcoma cell growth [70]. **(c)** MiR-193b can inhibit and repress TGFβ/SMAD and YAP pathways to regulate liposarcoma progression [30,98]. **(d)** Activated IL-6 pathway can promote nuclear translocation of NF-κβ and miR-25-3p and miR-92a-3p can upregulate IL-6 expression in TAM to promote IL-6 mediated oncogenic activity in liposarcoma cells [85]. **(e)** MiR-135 can suppress THBS2 to elevate the cellular expression of MMP2, promoting liposarcoma cell invasion and metastases [75]. The diagram was constructed using Biorender (https://app.biorender.com/gallery).

To date, over 15 miRNAs (Table 2; Fig. 1, Fig. 2e) have been documented to be involved in modulating the progression of liposarcoma. A combined clinical and *in vitro* study [34] has previously shown that miR-26a-2 was overexpressed (p < 0.05) by around 10-fold in several liposarcoma subtypes, including WDLPS, DDLPS, and myxoid/round cell liposarcoma. The increased expression of miR-26a-2 in liposarcoma patients was linked to decreased survival (p < 0.05) [34]. Upregulating this miRNA increased liposarcoma cell proliferation and migration *in vitro*, suppressing apoptosis and adipocyte differentiation [34]. The further functional assay identified that miR-26a-2 could inhibit the regulator of chromosome condensation and BTB domain-containing protein 1 (RCBTB1) to inhibit cancer cell apoptosis [34]. Re-expressing the protein made the cells more susceptible to cellular suicide [34]. RCBTB1 is a pro-apoptotic protein and cell cycle regulator critical in modulating sarcoma cell survival and chemoresistance [62]. Deletion of the gene encoding for this protein has been proven to contribute to accelerated sarcoma metastases and docetaxel resistance [62]. This explains why suppressing RCBTB1 by miR-26a-2 would promote liposarcoma development [34]. Two years later, the same group of researchers discovered that miR-26a-2 was overexpressed (p < 0.05) by about 10-fold in liposarcoma cell lines, and it could repress the expression homeobox protein 5 (HOXA5) *in vitro* [35]. Ectopic expression of HOXA5 then induced cancer cell apoptosis in a caspase-dependent and p53-independent manner [35]. HOXA5 has been documented to trigger caspase-mediated apoptosis and induce p53-independent apoptotic activities in solid tumours such as liposarcoma and breast cancer [63,64]. By inhibiting HOXA5 in liposarcoma cells, miR-26a-2 halted apoptosis, leading to uncontrolled cellular growth [35]. The findings from these two studies [34,35] consistently showed that miR-26a-2 is likely a tumour-promoting miRNA in liposarcoma, and it suppresses the expression of pro-apoptotic targets to inhibit cellular suicide. However, these findings were reported by a similar group of researchers. Hence, different groups of researchers should examine the tumour-promoting role of miR-26a-2 in liposarcoma to see if similar findings could be observed. In other cancer types, including breast cancer, miR-26a was found to block tumour cell growth and invasion by targeting tumour-promoting targets such as family with sequence similarity 98 member A (FAM98A) *in vitro* [65]. This finding suggests that miR-26a family members could play different tumour-modulatory roles in other types of cancer, and this needs to be verified further using future studies.

Casein kinase 1α (CK1α) is a multifunctional protein that regulates cell metabolism, differentiation, apoptosis, autophagy, and WNT signalling pathways [66,67]. Knockout of CK1α *in vivo* in mice was previously shown to induce the expression of tumour-promoting targets such as CD44 and cyclin D1 [66,68], suggesting CK1α plays an essential role in controlling the expression of multiple oncogenic targets. Dysregulation of cyclin D1 and WNT/β-catenin activities can then promote uncontrolled cell division and cancer metastases [66,69]. A study [70] has reported that miR-155 can accelerate liposarcoma development by inhibiting CK1α expression in liposarcoma cells (Fig. 2b) to increase the expression and activity of cyclin D1 and β-catenin. Measuring the expression of miR-155 in both WDLPS and DDLPS tissues and liposarcoma cell lines revealed that this miRNA is overexpressed (p < 0.05) by around 7.5-fold and 20- to 60-fold, respectively, in cancerous tissues and cancer cell lines [70], further indicating that it is a tumour-promoting miRNA. The tumour-controlling role of miR-155 has been widely reported [71,72]. In oral squamous cell carcinoma, miR-155 has been reported to downregulate B-cell leukemia 6 (BCL6) to increase the expression of cyclin D6 to enhance tumour proliferation and aggression [73]. On the contrary, miR-155 was shown to possess anti-tumour properties by inhibiting tumour cell growth and enhancing the influx of T cells into the tumour microenvironment to improve the anti-tumour immune environment [74]. The findings reported from various studies [[70], [71], [72],^74^] suggest that miR-155 is likely to exert different tumour-modulatory roles in various kinds of cancer, and the exact tumour-regulatory role of miR-155 in liposarcoma needs to be confirmed further as currently there are not many studies which have described its tumour-modulatory role in liposarcoma.

In another study [75] aimed at delineating the tumour-regulatory role of miR-135b, it was shown that this miRNA was upregulated (p < 0.05) by around 20-fold and 10- to 15-fold in both liposarcoma tissues and cell lines, respectively. MiR-135b can target and inhibit the expression of thrombospondin 2 (THBS2) to increase the invasion and migration processes in myxoid liposarcoma *in vitro* and *in vivo* [75]. Suppression of THBS2 led to increased expression of MMP2 (Fig. 2e) [75], suggesting a reciprocal regulatory mechanism between THBS2 and MMP2. THBS2 is known to regulate the expression of MMPs in human cells, such as cartilage cells [76,77]. As a protein that degrades tissue basement membrane, overexpression of MMP2 can facilitate cancer cell movement, leading to distant metastases and worsening disease [78]. In another previously reported study [79], THBS2 was reported to increase MMP expression rather than suppress their expression, and the finding contradicts the findings reported in the study by Nezu et al., which stated that THBS represses MMP expression. Therefore, a more detailed analysis should be performed to determine if regulating THBS expression by miRNA will increase or decrease MMP expression. On the other hand, knocking down miR-135b has been demonstrated to induce apoptosis in different tumour cells, including colorectal cancer cells [80]. Downregulating miR-135b also sensitized colorectal cancer cells to oxaliplatin, implying that the miRNA is likely a tumour-promoting miRNA [80].

MDM2 is a negative regulator of p53, and MDM2 is known to accelerate cancer development [81]. An *in vitro* study [82] has reported that miR-215-3p was upregulated (p < 0.05) 25-fold in liposarcoma cell lines, and this miRNA could increase MDM2 expression to decrease apoptosis activities in the liposarcoma cells (Fig. 2a) [82]. Subsequent immunoblotting assays showed that the expressions of pro-apoptotic proteins such as p53 and p21 were suppressed, and anti-apoptotic proteins like B cell leukemia 2 (BCL-2) were increased in the cells that overexpressed miR-215-3p, indicating this miRNA is a tumour-enhancing miRNA [82]. As this finding is merely based on cell-based assay findings, *in vivo* and clinical experiments should be conducted to confirm if miR-215-3p can increase MDM2 expression to accelerate liposarcoma development. In other solid cancers, such as colorectal and cervical cancer, miR-215-3p was reported to repress tumourigenesis than promote it [83,84]. For instance, miR-215-3p can suppress colorectal cancer growth and aggression by inhibiting Forkhead box protein M1 (FOXM1) expression [84]. In contrast, the same miRNA can inhibit cervical cancer proliferation and metastases by downregulating SRY-box transcription factor 9 (SOX9) expression [83]. As there is currently only a single study that has highlighted the tumour-accelerating role of miR-215-3p in liposarcoma, more study is needed to verify this finding [82] as the miRNA acts as a tumour-suppressor in other cancer types [83,84].

On the other hand, two miRNAs, namely miR-25-3p and miR-92a-3p, were demonstrated to be overexpressed (p < 0.05) by 2.5- to 3-fold in the extracellular vesicles released by liposarcoma cells [85]. Both miRNAs were found to stimulate the tumour-associated macrophages (TAM) to secrete pro-inflammatory cytokine, interleukin-6 (IL-6), which in turn promote liposarcoma cell growth, invasion, and metastases (Fig. 2d) [85]. In soft tissue sarcomas, elevation of IL-6 has been shown to correlate positively to decreased survival and poor clinical outcomes (p < 0.05) [86]. Overexpressing IL-6 can stimulate the activities of a few oncogenic pathways, including the nuclear factor-kappa beta (NF-Kβ) activities, leading to cancer progression and metastases [87]. To further confirm if exosomal miR-25-3p and miR-92a-3p enhance IL-6 secretion by TAM to promote liposarcoma progression, future studies can consider administering the inhibitors to these two miRNAs to see if this action can block IL-6 secretion by TAM and downregulate IL-6 signalling in the liposarcoma cells. Regarding the tumour-regulatory roles of miR-25-3p and miR-92a-3p, both miRNAs have been previously reported to promote cancer development [88,89]. For example, cancer-derived exosomal miR-25-3p has been shown to increase angiogenesis and vascular permeability in the colorectal tumour microenvironment to promote cancer metastases [88]. In lung cancer, silencing miR-92a-3p has been found to enhance tumour sensitivity to cisplatin, implying this miRNA is a tumour-promoting miRNA [89]. The collective findings reported in these two studies [88,89] support the observation seen in liposarcoma [85] in which the upregulation of exosomal miR-25-3p and miR-92a-3p could probably signify increased tumourigenesis.

### 4.2 Tumour-suppressing miRNAs in liposarcoma

In 2011, an integrated clinical and cell-based study [90] reported that miR-143 was downregulated (p < 0.05) by 3.3- to 7.9-fold and 10-fold in WDLPS and DDLPS clinical samples and liposarcoma cell lines, respectively. These observations implied this miRNA could be tumour-inhibiting in liposarcoma (Table 2). Functional assay revealed that miR-143 could repress the expressions of topoisomerase 2-alpha (TOP2A), protein regulator of cytokinesis 1 (PRC1), and polo-like kinase 1 (PLK1) to inhibit liposarcoma cell growth and trigger apoptosis [90]. TOP2A is involved in regulating DNA structural integrity, and its overexpression has been shown to be associated with poor survival among soft tissue sarcoma patients (p < 0.05) [91]. On the contrary, PRC1 can promote immune suppression in solid tumours such as liver cancer and contributes to genome instability that precipitates oncogenesis [92]. PLK1 is an oncogenic protein that enhances cell cycle progression and proliferation; inhibiting this protein can impede sarcoma growth effectively [93]. Considering the known tumour-promoting roles of TOP2A [91], PRC1 [92], and PLK1 [93], inhibiting these proteins by miR-143, therefore, helps inhibit liposarcoma progression [90]. A comprehensive review [94] has summarized that miR-143 is a potential tumour-repressing miRNA in various forms of cancer, and overexpression of miR-143 is known to correlate tightly with good clinical outcomes and improved survival rate among breast cancer patients [95]. However, as a single miRNA can target multiple downstream targets [96,97], and miR-143 has been shown to repress the expressions of three different targets [90], future study should be performed to see if miR-143 could also inhibit tumour-suppressing proteins as such action can possibly enhance cancer development.

Another study [98] discovered that miR-193b is a tumour-suppressing miRNA in liposarcoma as the expression of this miRNA was reduced significantly (p < 0.05) by 6- to 40-fold and 2- to 5-fold in DDLPS, WDLPS tissues, and liposarcoma cell lines, respectively. Combined transcriptomic and proteomic investigations revealed that miR-193b can target and inhibit CRK-like proto-oncogene (CRKL) and focal adhesion kinase (FAK) to slow liposarcoma development [98]. CRKL upregulation has been proven to activate the phosphoinositide 3-kinase (PI3K)/protein kinase B (AKT) pathway to accelerate solid tumour growth [99]. In contrast, increased FAK expression is known to promote the growth of sarcomas, including Ewing sarcoma and rhabdomyosarcoma [100]. In liposarcoma, miR-193b was found to suppress CRKL and FAK to induce apoptosis in liposarcoma cells, halting their growth *in vitro* and *in vivo* [98]. Two years later, the same group of researchers uncovered that miR-193b could also target platelet-derived growth factor receptor beta (PDGFRβ), suppressor of mothers against Decapentaplegic 4 (SMAD4), and yes-associated protein 1 (YAP1) to decrease liposarcoma cell growth and viability and induce adipogenic differentiation (Fig. 1, Fig. 2c) [30]. Activating PDGFRβ-mediated signalling activity is known to modulate the YAP transcriptional activity, and knockdown of PDGFRβ has been proven to decrease the expression of YAP target genes, leading to reduced carcinogenesis [101]. On the other hand, YAP can also act as the transcriptional regulator of the oncogenic WNT/β-catenin signalling pathway to promote tumour progression [102]. When activated, the WNT/β-catenin pathway can also crosstalk with the transforming growth factor-β (TGF-β)/SMAD pathway to accelerate tumourigenesis [103]. Considering the potential interaction among PDGFRβ, SMAD4, and YAP1 in precipitating cancer development [[101], [102], [103]], inhibiting these targets by miR-193b can therefore help halt liposarcoma progression [30]. Although miR-193b was reported to be a tumour-repressing miRNA in liposarcoma, the findings were reported by similar researchers [30,98] and raised a bias query. Hence, the tumour-regulatory role of miR-193b should be examined by different scientists to ensure its validity and precision. In other cancer types, such as ovarian cancer, miR-193b has been shown to possess a tumour-inhibiting role by targeting oncogenic proteins like stathmin 1 [104]. In contrast, miR-193b can also inhibit KRAS expression to block the development of oesophageal cancer [105]. These findings [104,105] suggest that miR-193b could play a tumour-repressing role in a few cancer types other than liposarcoma [30,98].

On the other side, miR-195 expression was found to be significantly (p < 0.05) lower by 2-fold in liposarcoma clinical samples and cell lines compared to normal cells [106]. Subsequent molecular analyses identified oxysterol-binding protein (OSBP) as the direct target of miR-195 and increased miR-195 expression inhibited (p < 0.05) liposarcoma cell growth and migration *in vitro* and *in vivo* [106]. Dysregulation of OSBP has been demonstrated to influence the survival of breast cancer patients [107], and OSBP-related proteins such as ORP2 have been recorded to increase EMT activities in cancers like pancreatic cancer to accelerate cancer progression [108]. As OSBP is likely a tumour-promoting protein, repressing its expression by miR-195 can slow liposarcoma development [106]. The tumour-regulatory role of miR-195 has been widely reported, and it can serve as a tumour-promoting or tumour-suppressing miRNA across multiple cancer types [109]. In lung and breast cancer [110,111], miR-195 was reported to play a tumour-repressing role, and its upregulation could link to improved prognosis among breast cancer patients [111]. In melanoma, miR-195 was identified as a tumour-promoting miRNA that promotes tumour growth, invasion, and migration by inhibiting WEE1 protein cellular expression, blocking cell cycle arrest, and enhancing uncontrolled cellular proliferation [112]. Because miR-195 could play various tumour-controlling roles in different cancer types, a more in-depth study needs to be performed to evaluate the putative tumour-regulatory role of miR-195 in liposarcoma.

Plasminogen activator inhibitor-1 (PAI-1) is a serine protease inhibitor involved in increasing cancer cell invasion and metastases, and silencing this protein is critical to slow cancer development [113,114]. MiR-486 was previously reported to inhibit PAI-1 expression in liposarcoma *in vitro* to inhibit cancer cell growth and repressing miR-486 elevated PAI-1 cellular level, helping to maintain liposarcoma cell survival [114]. Therefore, increasing miR-486 expression in liposarcoma could help prevent liposarcoma development by inhibiting the expression of PAI-1 [114]. However, future *in vivo* and human studies need to be conducted to validate if overexpressing miR-486 could repress PAI-1 in animal and human bodies to halt liposarcoma development. In other cancers, including ovarian and pancreatic cancers, miR-486 was reported to act as a tumour-promoting miRNA than a tumour-blocking miRNA [115,116]. For example, miR-486 was shown to accelerate cell cycle progression in ovarian cancer cells by increasing the expression of cyclin and cyclin-depending kinases [116]. In contrast, miR-486-5p was demonstrated to increase chemoresistance to 5-fluorouracil in pancreatic cancer cells [115]. The opposite findings on the tumour-regulatory roles of miR-486 in liposarcoma [114] and other solid cancers [115,116] warrant the need for a subsequent study to confirm the exact tumour-modulatory role of miR-486 in liposarcoma.

Hypoxia is known to promote cancer development, and aberrant expression of hypoxia-inducible factor 3 subunit alpha (HIF-3α) has been shown to accelerate cancer development and contribute to poor disease outcomes [117]. For instance, HIF-3α was demonstrated to promote pancreatic cancer metastases by upregulating the oncogenic RhoC-ROCK1 signalling pathway [118]. In liposarcoma, miR-210-3p and miR-485-5p were shown to target and inhibit HIF-3α expression *in vitro* to downregulate the oncogenic hypoxic-signalling activities [119], suggesting these two miRNAs could play tumour-inhibiting role in liposarcoma. A drawback of this study [119] is that the finding was based on *in vitro* findings without being backed by animal and clinical data. In glioma cells, miR-485-5p has been reported to repress the serine-arginine protein kinase 1 (SRPK1) to suppress tumourigenesis in hypoxia condition [120], consistent with the findings reported in the study involving liposarcoma that showed miR-485-5p also inhibited tumourigenesis by suppressing hypoxia-related activities [119]. Conversely, miR-210-3p expression has been linked to hypoxia conditions, and its expression could correlate positively with poor clinical outcomes and chemoresistance in breast cancer patients [121], raising a question of whether this miRNA could regulate hypoxia activities differently in liposarcoma [119] and other types of cancers [121]. On the contrary, another study by Gits et al. [33] showed that two other miRNAs, namely miR-145 and miR-451, were shown to have significantly lower expression (2- to 5-fold) in liposarcoma tissues compared to non-cancerous tissues. Overexpressing both miRNAs also impaired cell cycle progression, reduced cancer cell viability, and stimulated cellular suicide [33]. All these findings indicate that miR-145 and miR-451 are likely to serve as tumour-repressing miRNAs in liposarcoma, although the authors did not further discover their molecular targets in liposarcoma [33]. The direct targets of miR-145 and miR-451 in liposarcoma cells are worth exploring in future studies to unravel the molecular pathways of how these two miRNAs repress liposarcoma progression. In other studies, miR-145 and miR-451 were reported to mainly act as tumour-repressor across many cancer types [122,123]. For instance, miR-145 could target p70S6K1 to decrease the expressions of HIF and angiogenic factors located downstream to p70S6K1, thereby reducing tumourigenesis and angiogenesis [124]. MiR-451, on the other hand, could inhibit the malignant phenotypes of colorectal cancer cells by decreasing the expression of sterile alpha motif domain containing 4B (SAMD4B) to decrease carcinogenesis *in vitro* and *in vivo* [125]. These findings consistently support the fact that miR-145 and miR-451 could act as tumour-suppressing miRNAs in many cancers [[122], [123], [124], [125]], including liposarcoma [33].

Warburg effect states that glycolysis is enhanced, and oxidative phosphorylation is suppressed in many cancer cells compared to normal non-cancerous cells [126,127]. This phenomenon is suggested to be caused by permanent impairment of the oxidative phosphorylation activities in the cancer cell mitochondrion, and the effect is crucial to sustaining cancer cell survival and explaining cancer cell resistance to anti-cancer therapy [126,127]. A combined clinical, *in vitro*, and animal study [128] showed that miR-133a could suppress glycolysis and activate oxidative phosphorylation in DDLPS, reducing cancer cell proliferation and cell cycle progression (Table 2). Additionally, the expression of miR-133a was decreased prominently (p < 0.05) in liposarcoma tissues and cell lines by 40-fold and 2- to 5-fold, respectively [128], further highlighting it mainly acts as a tumour-suppressor in liposarcoma. However, the exact molecular mechanism of how miR-133a regulates glycolysis and oxidative phosphorylation in DDLPS is unclear, and this is worth to be explored in future studies. The tumour-inhibitory role of miR-133a in human cancer has been widely established [129,130]. For instance, miR-133a was reported to target cadherin 3 to repress colorectal cancer cell growth and aggression [130]. In contrast, the miRNA was shown to downregulate YES1 proto-oncoprotein expression to increase autophagy, inhibit ovarian cancer growth, and induce chemosensitivity to cisplatin [129].

## 5 Roles of lncRNAs in the progression of liposarcoma

### 5.1 Tumour-promoting lncRNAs in liposarcoma

Besides miRNAs (Table 2), several lncRNAs have also been shown to regulate liposarcoma development (Table 3 & Fig. 1). For instance, lncRNA TODL was reported to promote proliferation, cell cycle progression, EMT, and metastases in liposarcoma *in vitro* and *in vivo* [31]. This lncRNA was found to be overexpressed (p < 0.05) by around 5- to 20-fold and 80- to 180-fold in the clinical liposarcoma tissue samples and liposarcoma cell lines, respectively [31]. The upregulation of lncRNA TODL was shown to repress adipogenesis and differentiation in dedifferentiated liposarcoma, and silencing this lncRNA promoted adipogenesis and differentiation in dedifferentiated liposarcoma, slowing tumour progression [31]. Subsequent functional analyses discovered that lncRNA TODL would bind FOXM1, a critical oncogenic transcription factor and cell cycle regulator in human cells [31]. FOXM1 can promote cell cycle progression and pluripotency in sarcoma, accelerating sarcoma growth [131]. A recent study [132] has demonstrated that FOXM1 inhibitors can sensitive human cancer cells to various cancer therapies, further highlighting the tumour-promoting role of FOXM1. By stabilizing FOXM1, lncRNA TODL can promote cellular proliferation to enhance tumourigenesis in liposarcoma [31].

As a negative regulator of p53 and apoptosis pathway, dysregulation of MDM2 is known to facilitate cancer development and encourage cancer cell growth [81]. An integrated *in vitro* and *in vivo* study [27] has revealed that a lncRNA termed PILRLS can bind and stabilize T-cell leukemia/lymphoma protein 1A (TCL1A) in retroperitoneal liposarcoma cells to activate MDM2. Activating MDM2 resulted in the repression of the p53-mediated pathway, causing uncontrolled liposarcoma cell growth [27]. These findings suggested that PILRLS is likely to be a tumour-promoting lncRNA in liposarcoma, and the significantly higher expression (around 5-fold, p < 0.05) of PILRPS in the retroperitoneal liposarcoma tissues compared to the normal tissues further supported the tumour-accelerating role of PILRLS in the progression of liposarcoma [27]. In other cancer types, the tumour-regulatory role of lncRNA TODL and PILRPS is not widely reported. Therefore, more studies need to be conducted to determine whether lncRNA TODL and PILRPS could promote tumorigenesis in liposarcoma and other cancers, delineate the underlying mechanism of how this lncRNA regulates cancer progression, and find out whether this lncRNA could sponge any miRNA to control cancer development.

### 5.2 Tumour-suppressing lncRNAs in liposarcoma

In a study [28] involving retroperitoneal liposarcoma, LINC00423 was reported to act as a tumour-repressing lncRNA by downregulating the MAPK pathway (Fig. 1) via destabilizing nuclear factor of activated T cells 3 (NFATC3) (Table 3). The lncRNA was found to modulate the MAPK pathway at the transcriptional level negatively and destabilized NFATC3 at the post-transcriptional level [28]. Stimulating the MAPK pathway is known to increase the tumour staging and is associated with poor prognosis in liposarcoma such as dedifferentiated liposarcoma [133] while activating NFATC3 was previously found to promote the growth and migration of tumours such as glioma [134]. Considering the expression level of LINC00423 was reduced by around 1- to 2-fold (p < 0.05) in retroperitoneal sarcoma tissues compared to healthy tissues, and it suppressed the MAPK pathway and destabilized NFATC3 to halt liposarcoma growth *in vitro* and *in vivo*, LINC00423, therefore, is likely to play a tumour-repressing role in the progression of liposarcoma [28]. Like lncRNA TODL and PILRLS, the tumour-modulatory role of LINC00423 in human cancer is also not widely reported. However, a study [135] aimed at uncovering the dysregulated ncRNAs in osteosarcoma tissues revealed that LINC00423 was significantly upregulated (p < 0.05) in osteosarcoma tissues compared to adjacent bone tissues, suggesting the overexpression of this lncRNA was associated with osteosarcoma progression. Still, the study did not mention the molecular mechanism of how LINC00423 regulates osteosarcoma tumourigenesis [135]. Nevertheless, this discovery contradicts the previous observation that LINC00423 plays a tumour-repressing role by controlling the NFATC3 activity liposarcoma [28].

## 6 Diagnostic and prognostic significance of ncRNAs in liposarcoma

### 6.1 Diagnostic significance of ncRNAs

#### 6.1.1 Liposarcoma diagnosis using tissue and cellular ncRNAs quantification

**Fig. 3:**
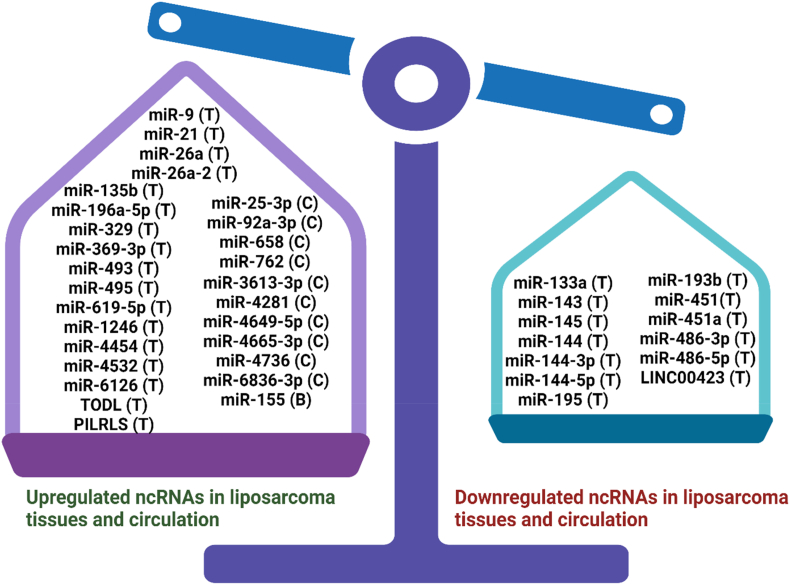
Summary of upregulated and downregulated ncRNAs in liposarcoma. A total of 28 ncRNAs were shown to be upregulated in liposarcoma tissues (T), circulation (C), or both (B) forms. In contrast, 11 ncRNAs were downregulated in the liposarcoma tissues. Examples of overexpressed miRNAs in liposarcoma tissues include miR-9, miR-21, miR-26a, miR-26a-2, miR-135b, miR-155, miR-196a-5p, miR-329, miR-369-3p, miR-493, miR-495, miR-619-5p, miR-1246, miR-4454, miR-4532, and miR-6126 [29,34,75,90,[138], [139], [140], [141]]. Upregulated circulating miRNAs in liposarcoma include miR-25-3p, miR-92a-3p, miR-155, and miR-3613-3p [85,142,143]. A serum miRNA panel known as index VI was reported to be useful in confirming the diagnosis of liposarcoma, and the panel includes miR-658, miR-762, miR-4281, miR-4649-5p, miR-4665-3p, miR-4736, and miR-6836-3p [146]. Downregulated miRNAs that can be found in liposarcoma tissues include miR-133a, miR-143, miR-145, miR-144, miR-144-3p, miR-144-5p, miR-193b, miR-195, miR-451, miR-451a, miR-486-3p, and miR-486-5p [29,30,90,106,128,139,141]. For lncRNAs, TODL and PILRLS are two overexpressed lncRNAs in liposarcoma tissues [27,31], whereas LINC00423 was found to be the underexpressed lncRNAs in liposarcoma tissues [28]. The diagram was constructed using Biorender (https://app.biorender.com/gallery).

Like many other cancer types, such as colorectal cancer [136] and breast cancer [137], miRNAs can also be potentially used as diagnostic biomarkers in liposarcoma (Table 4). To date, more than 20 miRNAs have been documented to be dysregulated in liposarcoma tissues (Fig. 3). Examples of the overexpressing miRNAs in liposarcoma tissues and their upregulation fold-change include miR-9 (2.8-fold), miR-21 (3.9-fold), miR-26a (3.2-fold), miR-26a-2 (∼10-fold), miR-135b (∼20-fold), miR-155 (8.7-fold), miR-196a-5p (unclear fold-change), miR-329 (2.8-fold), miR-369-3p, (2.9-fold), miR-493 (3.1-fold), and miR-495 (2.3-fold) [29,34,75,90,[138], [139], [140], [141]]. In liposarcoma cell lines, miR-26a-2 (∼10-fold), miR-155 (20-60-fold), miR-135b (∼10- to 15-fold), miR-619-5p (1.9- to 2.4-fold), miR-1246 (2.9- to 3.5-fold), miR-4454 (1.9- to 3.7-fold), miR-4532 (1.4- to 2.5-fold), and miR-6126 (0.6- to 0.8-fold) were also found to be upregulated (p < 0.05) *in vitro* compared to non-cancerous cell lines [35,70,75,140,141]. These findings were consistent with what had been observed in the liposarcoma clinical samples, making these miRNAs strong candidates to diagnose liposarcoma. On the contrary, examples of the underexpressing ncRNAs in liposarcoma tissues and their downregulation fold-change include miR-133a (40-fold), miR-143 (3.3–7.9-fold), miR-145 (∼10-fold), miR-144 (4.4-fold), miR-193b (6- to 40-fold), miR-195 (2.4-fold), miR-451 (2- to 5-fold), miR-486-3p (3.3-fold), miR-486-5p (3.9-fold), and LINC00423 (unclear fold-change) [[28], [29], [30],^90^,^106^,^128^,^139^,^141^]. In liposarcoma cell lines *in vitro*, miR-133a (∼2- to 5-fold), miR-143 (∼10-fold), miR-193b and (∼2-5-fold) and were also reported to be underexpressed that those measured in normal adipose cells [30,90,128], consistent with the trends observed in clinical liposarcoma tissues. The expressions of miR-210-3p and miR-485-5p were shown to be reduced (p < 0.05) in liposarcoma cell lines *in vitro* [119] (Table 2), but their expression pattern in clinical samples has yet to be clearly known. It is worth examining further if the tissue levels of these miRNAs are lower in liposarcoma tissues to decide if they can be potential diagnostic biomarkers in liposarcoma. To sum up, quantifying the tissue or cellular levels of these miRNAs could potentially help confirm the diagnosis of liposarcoma in addition to histological and molecular testing [10,29]. However, a validation study and subsequent clinical trials must be conducted to confirm the diagnostic accuracy, sensitivity, and specificity of these miRNAs.

**Table 4:** Diagnostic and prognostic significance of short and long non-coding RNAs in liposarcoma (n = 20).

miRNA	Tissue/circulating level of miRNA that suggests diagnosis (FC)	Prognostic Significance When Non-Coding RNA Level is High			References
		Differentiating various types of sarcoma/staging	Survival/Relapse	Treatment Resistance	
miR-9	High (Cancerous tissues) (2.8-fold)	NA	NA	NA	[138]
miR-26a-2	High (Cancerous tissues) (∼10-fold)	NA	Low	NA	[34]
miR-26a-5p	High (Cancerous tissues) (6.5-fold)	Differentiates DDLPS & other sarcoma subtypes	NA	NA	[150]
miR-155	High (Cancerous tissues) (∼1.5-5-fold)	Upregulated in all liposarcoma subtypes except WDLPS	NA	NA	[32]
miR-196a-5p	High (Cancerous tissues) (unclear FC)	Differentiates liposarcoma & lipoma	NA	NA	[29]
miR-135b	High (Cancerous tissues (∼20-fold) & cancer cell lines (∼10-15-fold))	NA	Low DFS	NA	[75]
miR-21	High (Cancerous tissues) (3.9-fold)	NA	NA	NA	[139]
miR-155	High (Cancerous tissues) (8.7-fold)	High	Low OS High relapse	NA	
miR-21 & miR-26a	High (Cancerous tissues) (3.2–4.1-fold)	NA	NA	NA	[90]
miR-15b-5p, miR-21-5p, miR-374a-5p & miR-454-3p	High (Cancerous tissues) (unclear FC)	Differentiates DDLPS & WDLPS	NA	NA	[29]
miR-17-5p, miR-20a-5p, miR-20b-5p, miR-93-5p, miR-106a-5p, miR-181a-5p, miR-193a-3p, miR-193b-3p, miR-365a-3p & miR-365b-3p	High (Cancerous tissues) (2.9–12.7-fold)	Differentiates myxoid liposarcoma & other sarcoma subtypes	NA	NA	[150]
miR-155	High (Plasma) (3.9-fold)	NA	NA	NA	[142]
miR-3613-3p	High (Blood) (∼3-fold)	NA	NA	NA	[143]
Index VI (miR-658, miR-762, miR-4281, miR-4649-5p, miR-4665-3p, miR-4736 & miR-6836-3p)	High (Serum) (1-2-fold)	NA	NA	NA	[146]
miR-25-3p & miR-92a-3p	High (Exosome) (2.5-3-fold)	NA	High (Recurrence)	NA	[85]
miR-619-5p, miR-1246, miR-4454, miR-4532 & miR-6126	High (Cancerous tissues, cell lines & exosome) (0.6–3.7-fold)	NA	NA	NA	[140]
miR-26a-2, miR-329, miR-369-3p, miR-493 & miR-495	High (Cancerous tissues) (2.2-3-fold)	Differentiates liposarcoma from non-tumour	NA	NA	[141]
miR-193b	Low (Cancerous tissues & cancer cell lines) (∼1.5-fold)	Differentiates DDLPS & WDLPS	NA	NA	[149]
miR-195	Low (Cancerous tissues) (∼2-fold)	NA	High	NA	[106]
miR-143 & miR-145	Low (Cancerous tissues) (3.3–7.9-fold)	NA	NA	NA	[90]
miR-193a-5p & miR-423-5p	Low (Cancerous tissues) (unclear FC)	Differentiates DDLPS & WDLPS	NA	NA	[29]
miR-143, miR-145 & miR-451	Low (Cancerous tissues) (3-6-fold)	NA	NA	NA	[139]
miR-144-3p, miR-144-5p & miR-451a	Low (Cancerous tissues) (unclear FC)	Differentiates liposarcoma & lipoma	NA	NA	[29]
miR-1, miR-133, miR-145 & miR-206	Low (Cancerous tissues) (11-100-fold)	Downregulated in WDLPS & synovial sarcoma	NA	NA	[151]
miR-21-3p, miR-29-3p, miR-221-3p & miR-7150	Low (Cancerous tissues) (2–5.7-fold)	Differentiates myxoid liposarcoma & other sarcoma subtypes	NA	NA	[150]
miR-144, miR-145, miR-195, miR-486-3p & miR-486-5p	Low (Cancerous tissues) (2.4–4.4-fold)	Differentiates liposarcoma from non-tumour	NA	NA	[141]
miR-215-5p	NA	NA	Low	NA	[82]
miR-26b, miR-27b, miR-106b, miR-195, miR-629, miR-1260 & miR-1274b	NA	NA	NA	High in eribulin non-responders	[154]
LINC00423	NA	NA	High	NA	[28]

NA: No information available.

#### 6.1.2 Liposarcoma diagnosis by measuring circulating ncRNA levels

Other than measuring tissue miRNA levels to support the diagnosis of liposarcoma, quantifying the circulating level of miRNAs could also help confirm the diagnosis of liposarcoma (Table 4). For example, plasma miR-155 level was significantly higher by 3.9-fold (p < 0.05) in DDLPS (n = 5) patients compared to healthy control (n = 10) [142]. In contrast, a significantly higher level (3-fold; p < 0.05) of miR-3613-3p was detected in the whole blood of DDLPS patients (n = 6) compared to healthy subjects (n = 4) [143]. Accumulating evidence has shown that exosomes carry essential information that can help diagnose cancer [144,145]. The exosomal levels of miR-25-3p and miR-92a-3p have been observed to be higher by 2.5- to 3-fold in liposarcoma tissues (n = 24) than those observed in adjacent normal tissues (n = 6) and upregulation of these two miRNAs was associated with poor prognosis with high disease recurrence rate (p < 0.05) [85]. On the other hand, miR-619-5p, miR-1246, miR-4454, miR-4532, and miR-6126 were found to be upregulated (p < 0.05) in the exosomes isolated from DDLPS patients (n = 17) compared to healthy subjects (n = 34) [140]. Additionally, the levels of these five miRNAs were also shown to be elevated in DDLPS tissues and liposarcoma cell lines [140]. These consistent findings highlight that measuring the levels of these five miRNAs in exosomes and tissues could confidently diagnose liposarcoma [140]. On the other hand, Index VI is a serum miRNA classifier that has been reported to help diagnose sarcoma, and the panel miRNAs include miR-658, miR-762, miR-4281, miR-4649-5p, miR-4665-3p, miR-4736, and miR-6836-3p [146]. Quantifying the serum level of these seven miRNAs could help diagnose liposarcoma as these miRNAs were overexpressed 1- to 2-fold in liposarcoma patients rather than in healthy control. Still, it might not confidently differentiate liposarcoma from other types of sarcomas, including chondrosarcoma and osteosarcoma [146], warranting further histological and molecular tests to confirm the diagnosis. Although circulating miRNAs have emerged as critical diagnostic biomarkers in confirming cancer cases [147,148], most of the discussed studies [85,140,142,143] only recruited a small number (n < 100) of liposarcoma and healthy subjects in assessing the diagnostic significance of the listed circulating miRNAs, raising queries of the reliability of these miRNA biomarkers. Therefore, more studies should be conducted to validate the sensitivity, specificity, and accuracy of these circulating miRNA biomarkers in diagnosing liposarcoma. Besides, more clarification and evidence are needed to confirm whether these miRNAs are released from the liposarcoma tumour sites or other body sites. This allows quantifying these miRNA levels to confirm liposarcoma diagnosis rather than another disease state.

### 6.2 Use of miRNAs to differentiate liposarcoma from non-cancerous tumours

Besides being useful in diagnosing liposarcoma, miRNAs can also differentiate liposarcoma from non-cancerous adipose tumours (Table 4). For example, upregulated miR-196a-5p and downregulated miR-144-3p, miR-144-5p, and miR-451a tissue levels could be used as a panel to differentiate liposarcoma (WDLPS (n = 24) and DDLPS (n = 20)) from lipoma (n = 34) [29]. However, the study did not mention the dysregulation fold-changes of these miRNAs [29]. Another genome-wide miRNA expression study [141] aimed at deciphering miRNA signatures that could be used to differentiate liposarcoma (DDLPS and WDPLS) (n = 25) from non-cancerous adipose tissues (n = 18) and lipoma (n = 3) showed that ten miRNAs were found to be differentially expressed (p < 0.05). Upregulated miRNAs in liposarcoma tissues include miR-26a-2, miR-329, miR-369-3p, miR-493, and miR-495, while downregulated miRNAs in liposarcoma tissues include miR-144, miR-145, miR-195, miR-486-3p, and miR-486-5p [141]. The upregulated miRNAs were overexpressed by 2.2- to 3-fold, whereas the underexpressed miRNAs were downregulated by 2.4- to 4.4-fold in liposarcoma tissues compared to the non-cancerous tissues [141]. The sensitivity and specificity of these miRNAs were 91 % and 97 %, respectively, with an AUC of 0.95. Hence, quantifying the tissue expression of these ten miRNAs could help in diagnosing liposarcoma from non-cancerous tissues [141]. As discussed in section 6.1, the two studies only used a relatively small sample size (n < 100) to determine whether the listed miRNAs can confidently differentiate liposarcoma from non-cancerous adipose tissues. Further study is needed to support the sensitivity, specificity, and validity of these miRNAs in differentiating liposarcoma from non-malignant adipose tissues to avoid misdiagnosis.

### 6.3 Use of miRNAs to differentiate various liposarcoma subtypes

**Fig. 4:**
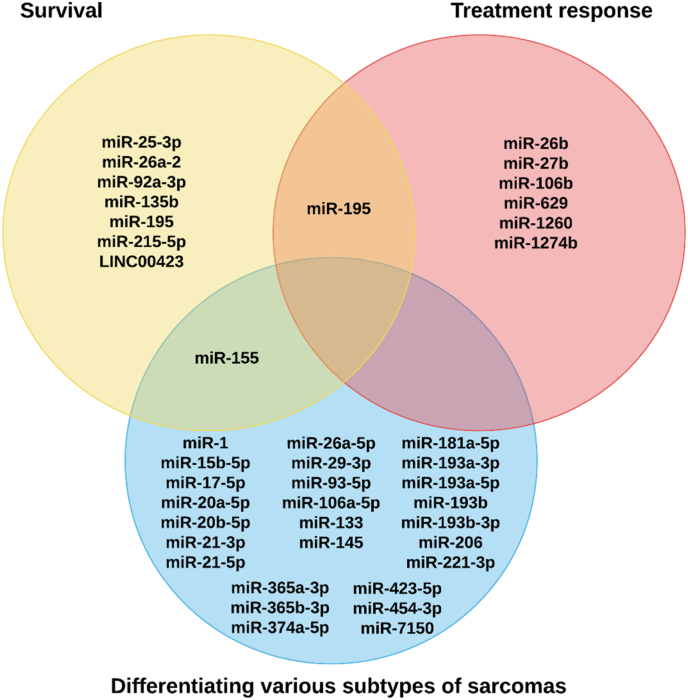
Classification of the roles of ncRNAs in differentiating various types of sarcomas, predicting survival rate, and treatment response. MiRNAs that can be used to differentiate DDLPS and WDLPS include miR-15b-5p, miR-21-5p, miR-374a-5p, miR-454-3p, miR-193a-5p, miR-193b, and miR-423-5p [29,149]. On the other hand, miR-26a-5p can be used to differentiate DDLPS from different subtypes of sarcoma [150], while miR-155 is the miRNA that was shown to be upregulated in all liposarcoma subtypes except WDLPS [32]. Yu et al. reported that miR-1, miR-133, miR-145, and miR-206 can differentiate WDLPS and synovial sarcoma from other sarcomas [151]. Besides, a study reported that a total of 14 miRNAs can be used to differentiate WDLPS from different types of sarcoma, and the miRNAs include miR-17-5p, miR-20a-5p, miR-20b-5p, miR-93-5p, miR-106a-5p, miR-181a-5p, miR-193a-3p, miR-193b-3p, miR-365a-3p, miR-365b-3p, miR-21-3p, miR-29-3p, miR-221-3p, and miR-7150 [150]. Regarding the potential to predict the survival of liposarcoma patients, overexpression of miR-26a-2 [34], miR-135b [75], miR-155 [139], and miR-215-5p [82] signify low survival rate, while upregulation of miR-25-3p, miR-92a-3p, miR-195, and LINC00423 signify high survival probability [28,85,106]. On the contrary, a miRNA panel measuring the expression of miR-26b, miR-27b, miR-106b, miR-195, miR-629, miR-1260, and miR-1274b can be used to predict the response of liposarcoma patients to eribulin [154]. Among all the listed miRNAs, miR-155 can be used in differentiating types of sarcomas and predicting survival [32,139], while miR-195 can be used to predict patient survival rate and eribulin response [106,154]. The diagram was constructed using Biorender (https://app.biorender.com/gallery).

Determining the histological subtypes of liposarcoma is crucial in evaluating patient prognosis and planning an appropriate treatment regimen [3,26]. Other than microscopic examination and molecular tests, measuring miRNA expression is now an alternative option to differentiate various liposarcoma subtypes (Table 4 & Fig. 4) [29,149]. A Singaporean study [29] involving 20 DDLPS and 24 WDLPS tissue samples discovered that four miRNAs, including miR-15b-5p, miR-21-5p, miR-374a-5p, and miR-454-3p were overexpressed (p < 0.05) in DDLPS compared to WDPLS [29]. In contrast, miR-193a-5p and miR-423-5p were downregulated (p < 0.05) in DDPLS tissues compared to WDLPS samples [29]. Measuring the tissue expression of these six miRNAs could help differentiate DDLPS and WDLPS [29]. However, the study did not mention the fold change of these dysregulations [29]. In another study, it was shown that the tissue miR-193b level was significantly lower by around 1.5-fold (p < 0.05) in DDLPS (n = 23) compared to WDLPS (n = 24) and normal adipose tissue (n = 11) [149]. This finding suggests that miR-193b expression could be silenced as the tumour progresses from a well-differentiated to a dedifferentiated state [149], making this miRNA a valuable biomarker to monitor disease progression. However, this speculation needs to be further verified using a longitudinal study investigating the progression of WDLPS to DDLPS, allowing researchers to understand how the expression of miR-193b changes dynamically as the disease progresses.

As a tumour-promoting miRNA in liposarcoma that activates the oncogenic β-catenin signalling pathway [70], miR-155 was detected to be upregulated by 1.5- to 5-fold (p < 0.05) in all liposarcoma subtypes, including DDLPS (n = 20), myxoid (n = 17), and pleomorphic liposarcomas (n = 5), except for WDLPS (n = 19) [32]. Hence, measuring tissue expression of miR-155 could help differentiate WDLPS from all liposarcoma subtypes [32]. In another clinical study [150] aimed at unveiling the specific miRNA signatures in different types of sarcomas, it was found that miR-26a-5p was upregulated by 6.5-fold (p < 0.05) in DDLPS (n = 9) from other sarcoma subtypes, making this miRNA a unique signature in confirming the diagnosis of DDLPS. The same study [150] also discovered 14 miRNAs that could be used to differentiate myxoid liposarcoma (n = 10) from other types of sarcomas (n = 35). Among these miRNAs, miR-17-5p, miR-20a-5p, miR-20b-5p, miR-93-5p, miR-106a-5p, miR-181a-5p, miR-193a-3p, miR-193b-3p, miR-365a-3p, and miR-365b-3p were upregulated by 2.9- to 12.7-fold (p < 0.05) in myxoid liposarcoma, whereas miR-21-3p, miR-29-3p, miR-221-3p, and miR-7150 were downregulated by 2- to 5.7-fold (p < 0.05) in myxoid liposarcoma compared to other subtypes of sarcomas [150]. On the other hand, miR-1, miR-133, miR-145, and miR-206 could potentially act as tumour-repressing miRNAs in WDLPS (n = 5) and synovial sarcoma (n = 5) as the expressions of these four miRNAs were found to be significantly lower by around 11- to 100-fold (p < 0.05) in these two sarcomas compared to other types of soft tissue sarcomas (n = 14) [151]. Histological and molecular diagnoses of various types of sarcomas can be challenging if they share highly similar mimicking features [61,152,153]. Using miRNA expression level can help diagnose and distinguish various types of sarcomas [10,150], allowing suitable treatment to be tailored to the patients. Currently, too many reported miRNAs can be potentially used to distinguish different soft tissue sarcomas. Besides the lack of validation studies to support the accuracy of these miRNAs, diagnostic costs and time can also increase if many miRNAs are chosen to confirm the diagnosis. Hence, there is a need to establish a specific miRNA panel that can quickly and accurately diagnose and differentiate various types of sarcomas, rather than having different miRNA panels, which can be confusing.

### 6.4 Prognostic significance of ncRNAs in liposarcoma

#### 6.4.1 Predicting patient survival

MiR-26a-2 has been demonstrated to serve as a tumour-promoting miRNA in liposarcoma [35]. Its overexpression was shown to be associated tightly with reduced survival (p < 0.05) in DDLPS and WDLPS patients (n = 72) compared to patients who underexpressed this miRNA (Table 4) [34]. In myxoid liposarcoma, increased expression of miR-135b (n = 14) has been shown to correlate positively (p < 0.05) to decreased disease-free survival (DFS) in patients compared to those who underexpressed miR-135b (n = 5) [75]. In another clinical study [139] involving 62 liposarcoma patients, it was demonstrated that upregulation of miR-155 was associated with lower overall survival and advanced disease staging (p < 0.05), implying that miR-155 could represent a novel independent indicator of poor prognosis in liposarcoma. Another miRNA that can predict low survival in liposarcoma patients is miR-215-5p, and this miRNA can increase MDM2 expression to suppress the p53 pathway, leading to uncontrolled cellular proliferation [82]. Compared to patients who underexpressed miR-215-5p (n = 29), patients with higher tissue levels of miR-215-5p (n = 29) were found to have a lower survival rate (p < 0.05) [82].

As a tumour-repressing miRNA that can suppress cellular proliferation and induce adipose cell differentiation, elevation of tissue level of miR-193b is linked to increased DFS (p = 0.015) in liposarcoma patients [98]. Besides miR-193b, the findings from a study involving 28 liposarcoma tissues demonstrated that a high tissue level of miR-195 could also signify a good prognosis and higher survival rate (p < 0.05) in liposarcoma patients compared to those who have low tissue level of miR-195 [106]. LINC00423 is a tumour-repressing lncRNA that downregulates the oncogenic MAPK pathway to slow liposarcoma growth [28]. Kaplan-Meier analysis that compared the survival rate of patients using 42 paired frozen retroperitoneal liposarcoma tissues with overexpressed and underexpressed LINC00423 showed that patients who overexpressed this lncRNA had a better survival rate (p < 0.05) [28]. Therefore, measuring the tissue expression of LINC00423 could help predict the survival rate of liposarcoma patients [28]. Although various ncRNAs can help predict the survival rate of liposarcoma patients, the sample size of most of the reported studies [28,34,75,85,106,139] was relatively small, or the studies lack validation cohort to support their prognostic significance and accuracy. Hence, all the discussed findings must be further verified using large-scale, multi-centric trial data to ensure the conclusions are reproducible and valid.

#### 6.4.2 Predicting treatment response

Regarding its role in predicting treatment response, miRNAs have also been reported to help predict the efficacy of anti-cancer treatment (Table 4 & Fig. 4). For example, tissue expressions of seven miRNAs, including miR-26b, miR-27b, miR-106b, miR-195, miR-629, miR-1260, and miR-1274b, were shown to be higher (p < 0.05) in eribulin non-responders than responders [154]. As eribulin is used in treating metastatic liposarcoma in patients who fail to respond to first-line chemotherapy [155], measuring the tissue expressions of these seven miRNAs could help predict whether liposarcoma patients are suitable for receiving eribulin [154]. A drawback of this study finding is that it only recruited 14 liposarcoma patients [154], and a big-scale clinical study involving patients from different age groups, races, and regions should be conducted to validate the accuracy of this miRNA panel in predicting eribulin response among liposarcoma patients.

## 7 Therapeutic potential of ncRNAs in liposarcoma

Theoretically, inhibiting tumour-promoting ncRNAs and overexpressing tumour-repressing ncRNAs can help eradicate cancer [[156], [157], [158]]. Over 15 ncRNAs have been reported to regulate the expressions of various targets in controlling the progression of liposarcoma (Table 2, Table 3). By restoring the expression of tumour-suppressing ncRNAs [28,30] and reducing the expression of tumour-promoting ncRNAs [31,75], it is possible to halt the progression of liposarcoma. Before ncRNAs can be used to treat liposarcoma, several problems need to be addressed. First, ncRNAs like miRNAs and lncRNAs can target multiple targets, leading to possible unwanted off-target effects [159]. For instance, miR-193b was reported to target various tumour-promoting targets to inhibit liposarcoma growth [30,98]. However, miR-193b was also found to promote tumour-progression in other types of cancers like head and neck cancer by blocking the activity of tumour-repressing protein [160]. Hence, it is vital to check and confirm that the ncRNA-based therapeutic strategy will not enhance cancer development by silencing tumour-suppressing targets. Secondly, delivering small molecules that mimic ncRNAs or ncRNA inhibitors into the human body for therapeutic purposes is not easy as oral administration may predispose them to acid digestion, and intravenous administration can cause their rapid degradation by nuclease [159,161]. Hence, choosing a safe and suitable carrier and delivery method is essential to ensure the nucleic acid therapeutic agent can be delivered specifically to the tumour site without causing toxicities and other side effects [159,161]. Database search using the clinical trial registry (https://clinicaltrials.gov/) (Date assessed: July 29, 2024) showed that no miRNA or lncRNA and associated molecules are currently used in clinical trials to eradicate liposarcoma. As substantial evidence [82,85] has shown that targeting ncRNAs could help eliminate liposarcoma, clinical trials should be initiated to see if ncRNAs are effective in killing liposarcoma cells *in vivo* in the human body without affecting normal functioning cells or causing toxicities.

## 8 Limitations and future implications for translational applications

Based on the findings from over 25 original research articles, nearly 40 different ncRNAs were discussed regarding their tumour-regulatory roles and translational potential in liposarcoma. Except for a few miRNAs such as miR-21 [90,139], miR-155 [32,70], and miR-195 [106,154], most of the tumour-regulatory roles or translational potentials of other ncRNAs were based on the findings from a single study, raising the reliability and reproducibility of the reported findings. Although the tumour-modulatory and translational potentials of miR-26a-2 [34,35] and miR-193b [30,98] were based on the findings reported in two studies. Still, the findings were reported by a similar group of researchers, raising a biased concern about the study's validity. Additionally, most of the discussed studies employed a relatively small sample size (n < 100) when evaluating the diagnostic and prognostic potentials of different ncRNAs involving human samples [75,106,139,150,154]. To further evaluate and confirm the translational values of the discussed ncRNAs, more studies, exceptionally detailed mechanistic studies, and multi-center trials with large sample sizes should be conducted by different groups of researchers to assess if the discussed ncRNAs can be used as biomarkers or therapeutic agents.

Next, miR-155 was found to be overexpressed by around 8.7-fold in liposarcoma tissues, and its overexpression could link to poor overall survival and signify a high risk of relapse [139]. Additionally, miR-155 was also shown to be highly expressed by 3.9-fold in the plasma of the liposarcoma patients [142], making this miRNA an excellent candidate to detect liposarcoma and predict the clinical outcome of liposarcoma patients. On the other hand, miR-195 was shown to be underexpressed by around 2-fold in liposarcoma tissues [106], and its overexpression could suggest a poor response to eribulin in liposarcoma patients [154]. Given too many original articles have reported different ncRNAs that could be potentially useful to be employed as liposarcoma biomarkers or therapeutic agents, it is impractical to further the investigation on all the discussed ncRNAs to confirm their translational values as this is time- and cost-consuming. Instead, future studies could focus on a few selective ncRNAs like miR-155 and miR-195 to further evaluate whether they could pose promising values as biomarkers, therapeutic agents, or targets. Compared to a single biomarker, the use of a multianalyte biomarker panel is said to have superior and better sensitivity in detecting cancer [162,163], and miR-155 and miR-195 could be a good pair as a liposarcoma biomarkers. Suppose these miRNAs could be proven to have translational values as biomarkers. In that case, further work should be carried out to see if manipulating the tissue levels of these miRNAs could achieve therapeutic potential. For instance, if miR-155 could be proven as a tumour-promoting miRNA that is overexpressed in the liposarcoma tissues, future works should focus on whether knocking down this miRNA could halt liposarcoma progression. This should involve designing and planning experiments concentrating on the appropriate way to deliver the miRNA to the cancer site to achieve its therapeutic goal and delineating the molecular mechanism of how the miRNA poses an anti-cancer effect *in vivo* in humans. If the preclinical work is successful, future trials involving liposarcoma patients should be conducted to confirm the safety, efficacy, and effectiveness of such a nucleic acid-based therapeutic approach.

## 9 Conclusion

This review summarizes the findings from over 25 original research articles on the tumour-regulatory roles and translational potentials of more than 30 miRNAs and lncRNAs in liposarcoma. By regulating the expressions and activities of various cellular targets such as BCL-2, cyclin D, PDGF, YAP, and IL6, at least 15 ncRNAs can regulate the activities of apoptotic and oncogenic WNT/β-catenin, TGF-β/SMAD4, EMT, interleukin, and YAP-associated pathways in liposarcoma to modulate cellular growth and metastases. Subsequently, this would affect liposarcoma progression. Regarding tissue and circulating expression, 28 ncRNAs were upregulated in liposarcoma cells or circulation, whereas 11 ncRNAs were downregulated, making them potential diagnostic biomarkers for liposarcoma. Besides, measuring the tissue or circulating levels of some of these ncRNAs could also help differentiate liposarcoma from benign tumours or other types of sarcomas and to predict the survival rate and clinical outcomes of liposarcoma patients. Examples of these miRNAs include miR-155, which can be used to differentiate liposarcoma subtypes and predict patient survival, whereas miR-195 can be employed to predict patient survival and eribulin response, making these two miRNAs valuable prognostic biomarkers. Although multiple ncRNAs have translational potential to be used as biomarkers in managing liposarcoma, most of the reported findings were derived from a single study with a relatively small sample size, raising data reproducibility and reliability concerns. Therefore, the accuracy, sensitivity, and specificity of most of these ncRNAs as biomarkers still need to be further verified. At the same time, it is impractical to further investigate the translational potentials of all the discussed ncRNAs, as this can be costly and time-consuming. Instead, it is more practical to evaluate further the translational potentials of a few selected miRNAs like miR-155 and miR-195 in which at least two different studies by different groups of researchers have shown that both miRNAs have translational values in diagnosing or prognosing liposarcoma cases. Regarding the therapeutic potential of ncRNAs in managing liposarcoma, no trial has been registered to evaluate the therapeutic potential of ncRNA-based therapeutic agents, and issues like the efficacy and safety of such therapeutic approaches need to be carefully examined before they can be tested in the human body to avoid unwanted side effects. On the other hand, compared to miRNAs and lncRNAs, there is a lack of a report highlighting the tumour-regulatory roles of other types of ncRNAs, like circRNAs. Therefore, it will be interesting if RNA researchers can explore and discover if circRNAs can regulate liposarcoma progression and see if such circRNAs can be biomarkers in managing liposarcoma.

## CRediT authorship contribution statement

**Zhi Xiong Chong:** Writing – review & editing, Writing – original draft, Visualization, Validation, Software, Resources, Project administration, Methodology, Investigation, Formal analysis, Data curation, Conceptualization. **Wan Yong Ho:** Writing – review & editing, Writing – original draft, Visualization, Validation, Supervision, Investigation, Conceptualization. **Swee Keong Yeap:** Writing – review & editing, Writing – original draft, Visualization, Validation, Supervision, Investigation, Conceptualization.

## Ethical approval

Non-applicable.

## Financial and non-financial competing interests declaration

The authors declare that they have no competing interests.

## Funding

This article is not funded by any agency or organization.

## Declaration of competing interest

The authors declare that they have no known competing financial interests or personal relationships that could have appeared to influence the work reported in this paper.
